# Discrete call types referring to predation risk enhance the efficiency of the meerkat sentinel system

**DOI:** 10.1038/srep44436

**Published:** 2017-03-17

**Authors:** R. Rauber, M. B. Manser

**Affiliations:** 1 Animal Behaviour, Department of Evolutionary Biology and Environmental Science, University of Zurich, Winterthurerstrasse 190, 8051, Zurich, Switzerland; 2 Kalahari Meerkat Project, Kuruman River Reserve, Northern Cape, South Africa; 3 Mammal Research Institute, University of Pretoria, Pretoria, South Africa

## Abstract

Sentinel behaviour, a form of coordinated vigilance, occurs in a limited range of species, mostly in cooperative breeders. In some species sentinels confirm their presence vocally by giving a single sentinel call type, whereby the rate and subtle acoustic changes provide graded information on the variation of perceived predation risk. In contrast, meerkat (*Suricata suricatta*) sentinels produce six different sentinel call types. Here we show that manipulation of perception of danger has different effects on the likelihood of emitting these different call types, and that these call types affect foraging individuals differently. Increasing the perceived predation risk by playing back alarm calls decreased the production rate of the common short note calls and increased the production rate of the rare long calls. Playbacks of short note calls increased foraging behaviour and decreased vigilance in the rest of the group, whereas the opposite was observed when playing long calls. This suggests that the common call types act as all-clear signals, while the rare call types have a warning function. Therefore, meerkats increase the efficiency of their sentinel system by producing several discrete call types that represent changes in predation risk and lead to adjustments of the group’s vigilance behaviour.

Accurate risk assessment is important for every animal living in an environment with unpredictable levels of predation risk^1^,^2^. In group-living species, public information, information that is obtained by monitoring other group members’ interactions with their environment, plays an important role in the assessment of predation risk^3^,^4^. Many species use alarm calls to warn other group members about immediate threats, which typically elicit appropriate responses, interrupting their foraging and for example running for shelter^5^,^6^,^7^. Variation in alarm calls of a vast number of different species are known to contain information on the type of threat^5^,^8^, predator size^9^, distance^10^, location^11^,^12^, or the type and urgency of a flight response^6^. Due to the costs involved in these responses, information about changes in predation risk before an immediate response is required seems beneficial. It allows receivers experiencing different risk levels, e.g. due to the variation of how close an individual is to shelter, or their investment into pursuing a specific prey item, to adjust their vigilance and foraging behaviour accordingly^13^,^14^. A limited number of species, typically cooperative breeders, such as the dwarf mongoose^15^ (*Helogale parvula*), meerkats^16^, or the pied babblers^17^,^18^ (*Turdoides bicolor*) evolved a sentinel system, a form of coordinated vigilance behaviour, where one individual is on guard while the rest of the group is involved in other activities, mainly foraging^13^,^19^,^20^. In species, where dense vegetation or foraging strategies prevent the visual location of other group members, sentinels emit the Watchman’s song^21^, soft vocalisations of low amplitude, to announce their presence. All detailed studies of sentinel vocalisations found that sentinels give a single, potentially graded (with a continuous change along one or several acoustic parameters), type of sentinel call (reviewed in ref. 20), whereby the rate of these calls seems to provide information on the variation of the perceived predation risk^14^,^22^. Here we show that meerkat sentinels produce a variety of different call types, besides emitting alarm calls when predators approach^16^,^23^.

Meerkats are a small, highly cooperative mongoose species that occur in arid, semi-desert areas of southern Africa and live in groups from three to 50 individuals^24^,^25^. They are diurnal and spend most of their time foraging for invertebrates and small vertebrates^24^, often moving within a distance of 20–50 meters from the closest shelter^26^,^27^. During foraging, meerkats mainly dig for their prey in the sand, which prevents them from visually scanning their surrounding for predators, such as martial eagles (*Polemaetus bellicosus*), jackals (*Canis mesomelas*) and caracals (*Felis caracal*)^6^,^28^. These factors together, namely the open environment, the inability to scan it during foraging and the variety of predators, resulted not only in the evolution of functionally referential alarm calls, containing information about both the predator type and the urgency level^6^,^29^, but also in the evolution of sentinel behaviour to coordinate group vigilance and hence optimize the foraging-vigilance trade-off for the whole group^23^. Meerkats on guard produce at least six different call types of soft calls during the entire length of the guarding session (Fig. 1)^23^. Most of these calls, especially the short note calls are also given in other contexts, such as foraging, or sunning^30^. However, the functions of these different call types in the sentinel context and the ultimate reason for this diversity in sentinel call types to evolve are still unknown.

We investigated the functions of the different meerkat sentinel call types in regards to public information about perceived predation risk. Based on previous work on meerkat sentinel vocalisations^23^, we grouped four of the six known call types into two different contextual categories. The most frequently produced “single-” and “double note” calls were grouped together into a ‘tonal short note calls’ category (hereafter short note calls) and the rare “di-drrr” and “wheek” calls into a ‘modulated long calls’ category (hereafter long calls) (Fig. 1), hypothesising that the former have a calming and the latter a warning function for the receivers of the signal. In order to understand the function of acoustic signals in animals, it is important to consider not only the contexts in which they are produced, but also the information receivers extract from these signals^31^,^32^. We tested whether changes in the perceived predation risk, induced by playing back meerkat alarm calls had an influence on call rate or call type emitted by the sentinel, similar to playback experiments performed with dwarf mongooses^22^. We expected sentinels to adjust their vocalisations according to the perceived predation risk in terms of decreasing the rate of short note calls while at the same time increasing the rate of long calls with an increase in perceived predation risk. We then investigated the receiver side by analysing how foraging test subjects responded to sentinel calls from the two different categories focusing on foraging and vigilance behaviour. We expected the two sentinel call categories to contain different information about the temporary perceived predation risk and hence to lead to a corresponding change in the vigilance behaviour of the foraging group, i.e. a decrease in vigilance after playbacks of short note calls and an increase in vigilance after the playbacks of long calls.

## Results

### Influence of changes in predation risk on sentinel vocalisations

Playing back alarm calls to the sentinel in order to increase the perceived predation risk had different effects on the production rate of the different sentinel call types. Meerkat sentinels decreased the rate of short note calls (LRT; 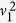 = 5.41, df = 1, p = 0.025; Fig. 2a) and at the same time increased the rate of long calls (LRT; 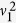 = 10.03, df = 1, p = 0.002, Fig. 2b) within 30 seconds after the playback of an alarm call compared to the time analysed before the playback. When the entire recording period of five minutes after the playback was taken into account, there was no effect of the alarm calls on the production rate of short note calls (LRT; 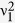 = 3.341, df = 1, p = 0.067) or long calls (LRT; 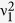 = 0.747, df = 1, p = 0.387).

### Influence of different sentinel call categories on foraging group members

Testing the response of foraging subjects within 30 seconds after a playback showed that different playback conditions (i.e. short note calls, long calls, and the two control conditions: close calls and background noise), had significant overall impacts on all of the measured behaviours: foraging (LRT;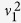 = 39.36, df = 2, p < 0.001), scanning (LRT; 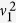 = 24.58, df = 2, p < 0.001) and bipedal scanning (LRT; 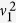 = 925.34, df = 2, p < 0.001). Behavioural responses did not differ significantly between the playbacks of close calls and background noise for any of the analysed behaviours (Table 1; Fig. 3), validating the use of close calls as control condition that not any soft call might have a calming or warning effect.

For the foraging behaviour, we found that meerkats spent more time foraging during the playback of short note calls compared to playbacks of close calls and long calls (Table 1; Fig. 3a). Considering close calls and long calls, meerkats spent more time foraging during the playback of close calls compared to long calls (Table 1; Fig. 3a). Focusing on vigilance behaviour, meerkats spent about the same amount of time scanning their surrounding quadrupedal during the playback of short note calls and close calls (Table 1; Fig. 3b). However, in the context of long calls being played back, meerkats showed a significant increase in quadrupedal scanning compared to both short note calls and close calls (Table 1; Fig. 3b). A similar pattern was found for the second type of vigilance we focused on, bipedal scanning, where meerkats spent about the same amount of time on bipedal scanning during the playback of short note and close calls (Table 1; Fig. 3c). But as soon as long calls were being played back, there was a significant increase in bipedal scanning compared to both short note calls and close calls (Table 1; Fig. 3c).

## Discussion

Our study provides evidence that meerkat sentinels alter their vocalisations based on temporary changes in the perceived predation risk and that foraging group members use this information to adjust their own vigilance and foraging behaviour accordingly. In contrast to other species using only one, potentially graded, call type as Watchman’s song, meerkats seem to increase the efficiency of their sentinel system, by producing additional types of sentinel calls (i.e. long calls) that indicate an increase in perceived predation risk, but no need for an immediate flight response, which allows the rest of the group to continue foraging.

### Production of different sentinel call categories depending on predation risk

Increasing the potential predation risk, by playing back alarm calls, affected the likelihood of the production of the two categories of sentinel call types in opposite directions. Sentinels decreased the rate of short note calls and at the same time increased the rate of long calls after hearing an alarm call. The decrease in the rate of short note calls provides evidence that these calls (“single-” and “double note” sentinel calls) act as all-clear signals, which have also been referred to as the Watchman’s song^21^, similar to what is known for sentinel calls of reed buntings^33^ (*Emberiza schoeniclus*), dwarf mongooses^22^ and pied babblers^34^. In these species, a higher call rate refers to low predation risk, while a decrease in call rate or the eventual absence of the calls, signals an increase in the temporal predation risk and leads to behavioural adjustment of the groups own vigilance behaviour^13^,^14^,^22^,^35^. This is in accordance with our findings that the playback of an alarm call, which was assumed to represent an increase in the perceived predation risk in the sentinel by simulating the presence of a predator, led to a decrease of this signal.

The increase in the rate of long calls after the playback of alarm calls provides evidence that “di-drrr” and “wheek” sentinel calls have a warning function and are mainly given after an increase in the perceived predation risk. Contrary to species using only one type of vocalisation as watchman’s song^21^,^22^,^33^,^34^, meerkat sentinels not only decrease in the rate of short note calls, but additionally emit long calls. Thereby, they specifically inform the group about an increase in the temporary predation risk, as well as the fact that there is still a sentinel on guard, which would give the appropriate alarm calls if a flight response of the rest of the group was needed.

The disadvantage of a sentinel system in which the decrease of the Watchman’s song, or even silence, in extreme cases^35^, signals an increase in predation risk is that this information could mistakenly be interpreted as the absence of a sentinel. Hence, the rest of the group has to respond in any case: either they have to react to the potential threat detected by the sentinel or they have to increase their vigilance as response to the finished guarding bout. However, foregoing foraging opportunities when there is no urgent threat has costs in terms of a reduced food intake, which would be even higher in harsh environments with food being rare or hard to extract. For meerkats, which live in a harsh environment, this problem seems to be solved by the presence of additional warning call types produced by sentinels. Therefore, this additional call category, which has not been described before in any other sentinel system, seems to further increase the efficiency of information transfer between the sentinel and its group in order to maximise food intake and hence optimize the trade-off between foraging and vigilance. At the same time this brings up the question why other species, such as pied babblers, a cooperative breeding bird living in similar environmental conditions do not show this differentiation in sentinel calls and what is the ultimate cause for the diversity in discrete sentinel calls in meerkats.

These effects of an increase in the perceived predation risk on the rate of short note and long calls, however, were only present within a short time period of 30 seconds after the playback, but not when taking the longer time period of five minutes into account. This can be explained by previous findings, which demonstrate that the time it takes for meerkats to relax after hearing alarm calls is on average no more than 60 seconds^36^. This reflects the need of a quick recovery after a predator incident in order to keep a positive balance towards acquiring food versus avoiding predation in an environment in which high fluctuations in predation risk are frequent.

### Response of foraging group members to different sentinel call categories

Foraging test subjects responded very differently to the playbacks of the two different sentinel call categories. Playing back short note calls led to less vigilance behaviour and more time invested in foraging. This confirms the findings of a decrease of alertness in foraging group members when playing back sentinel calls, of which the short note calls are the most frequently emitted calls^23^. It supports our hypothesis that short note calls act as an all-clear signal and are therefore very similar to sentinel calls referred to as Watchman’s song in other species. Playing back long calls, on the other hand, led to a general increase in vigilance behaviour in foraging test subjects. This confirms that long calls function as pre-stage of alarm calls, leading to receivers being on average more vigilant, but also informing them that there is no need for an immediate flight response. This enables group members that have already invested energy by digging a hole to extract food to be able to continue foraging. These long calls hence further increase the efficiency of the sentinel system by having a call, which is given when the predation risk increases and therefore enables the group to adjust their own vigilance behaviour accordingly. At the same time, long calls imply that the sentinel is still on guard, which allows the group to continue foraging despite higher vigilance levels rather than all of them interrupting foraging, as they typically do when they hear an alarm call^36^. These findings provide further evidence that short note calls function as an all-clear signal, while long calls have an alerting function and that the receivers of the signal use this information to adjust their behaviour according to the temporary predation risk.

### Conclusions

Our study provides evidence that meerkat sentinel calls convey public information about subtle changes in the current predation risk, which then leads to behavioural adjustment in the rest of the group. We suggest the use of the terms ‘calming calls’ and ‘warning calls’ for the two discrete and functionally different categories of sentinel calls. While meerkat sentinels optimize the trade-off between foraging and avoiding predation with multiple distinct call types, other species express variation in predation risk in changes of call rate only. This brings up the question under what circumstances discrete vocalisations are of advantage, and when graduation of a single call type by means of variation in call rate is sufficient for the same function. Further research needs to investigate how the remaining two sentinel calls (“triple-” and “multiple note” calls) fit into the context of calming and warning calls and, potentially, whether the call order of specific sentinel calls provide the group with valuable information about changes in the predation risk. This would shed light into how discrete and graded the meerkat sentinel calls are and how flexibly these vocalisations are used. Vocal flexibility and the importance of social learning in vocal communication in mammals is still a controversial topic^37^. However, our study is in accordance with the increasing evidence that animals are capable of voluntary control of the onset and offset of vocalisations, known as contextual learning^37^,^38^,^39^, in order to optimize the trade-off between foraging and antipredator behaviour.

## Methods

### Study site and species

The study was conducted between May and November 2014 at the Kuruman River Reserve in the southern Kalahari Desert, South Africa (for more information about habitat and climate at the study site see refs 28 and 40. As part of the Kalahari Meerkat Project’s long term data collection, all group members were uniquely dye marked to allow individual recognition, and one or two individuals of each group were fitted with radio-collars to facilitate localisation of the group (see ref. 41 for details of capture and collaring procedures). We conducted playback experiments in a total of nine groups with group size of eight to 24 individuals. All groups were habituated to close human observations and to the sound recording and playback equipment, allowing us to perform the recordings within a distance of 0.2–0.5 m from the calling meerkat.

### Sound recordings

All recordings from sentinel vocalisations were done using a Sennheiser directional microphone (ME66/K6) connected to a Marantz PMD-670 solid-state recorder (Marantz Japan Inc.; sampling frequency 44.2 kHz, 16 bits accuracy). A Rainhardt microphone windshield (W200) was permanently attached to the microphone to ensure high quality ‘recordings in the meerkats’ natural environment. Whenever the sentinel was calling from a tree or any other position difficult to access, the microphone was fixed to a telescopic pole in order to maintain the recording distance of less than 0.5 meters and thereby maintaining a high signal-to-background ratio.

### Playback experiments

To edit the sound files for the playback experiments, we used Cool Edit Pro (Syntrillium Software Corporation) to select single calls with a high signal-to-noise ratio. To play the selected calls back, we used a Marantz PMD-670 solid-state recorder connected to an iHome rechargeable mini speaker (iHM79SC). The amplitude was assessed according to how the calls occur under similar natural weather and wind conditions. Playback experiments were only conducted when no predator had been seen for at least five minutes and only if the group was foraging normally.

### Experimental set-up and observations

#### Influence of alarm calls on sentinel vocalisations

To measure the influence of an increased level of predation risk on sentinel vocalisations, playback experiments of a wide range of alarm calls, i.e. low and high urgency alarm calls for aerial and terrestrial predators, were conducted. The 15 unique alarm calls chosen for the playbacks were recorded from different individuals from the same meerkat population during previous years, as it is known that meerkats do not differentiate between alarm calls of their own group compared to alarm calls from a foreign group^42^. Each playback consisted of one alarm call bout (5–30 alarm calls given shortly after each other) of three to ten seconds and each alarm call was used a maximum of three times and always in different groups. For each of our eight groups we played a total of 4 alarm call playbacks (aerial high, aerial low, terrestrial high and terrestrial low), resulting in a total sample size of 32 playbacks. To investigate the effect of alarm calls being played back to individuals on guard, we recorded sentinel vocalisations for two minutes prior to the playback and five minutes after the playback. The distance between the speaker and the sentinel on guard was at least seven meters while the distance between the sentinel and the microphone was 0.3–0.5 m. As alarm calls are given quite frequently under natural conditions (on average every 45 minutes)^6^, it was possible to conduct two alarm playbacks during the same foraging session over typically 3 hours, but with at least a 30 minutes break in between, without risking subjects habituating to the playback procedure.

#### Influence of different sentinel call categories on foraging group members

To investigate the information group members extract from the two different categories of sentinel vocalisations (short note and long calls) we compared the behavioural response of foraging group members to playbacks of these two call categories, focusing on two behaviours: foraging and vigilance. The playbacks were conducted on four adult individuals (>12 months) per group, resulting in a total sample size of 32 playbacks. One playback file was composed of five minutes of short note calls (“single note” and “double note” sentinel calls) and another five minutes of long calls (“di-drrr” and “wheek” sentinel calls) of the same individual. Before, between these two categories and afterwards five minutes of close calls (cc) from the same individual were played back. This resulted in playbacks of a length of 30 minutes each (e.g. cc-short note-cc-long calls-cc-background). Close calls (cc) are the most commonly emitted vocal signals used by the meerkats for group coordination while foraging^43^ and were used as control condition. At the beginning or end of each playback, another five minutes of background noise were added to control for the impact of any call being played back and hence to check the utility of close calls as control to demonstrate baseline behaviour. To avoid any order effects, the order of short note calls and long calls was alternated between the different playbacks. The rates of the close calls and short note sentinel calls were kept the same for the playbacks as calculated from the natural recordings with background noise between each call (close calls: 8.25 ± 2.28 calls/min; single note calls: 3.79 ± 0.43 calls/min; double note calls: 3.19 ± 0.37 calls/min). For the long calls context we always played a total of four calls, two “di-drrr” and two “wheek” calls in alternating order and with at least one minute of background noise in between, which also lies in the range of natural recordings (di-drr: 0.34 ± 0.12 calls/min; wheek calls: 0.39 ± 0.09 calls/min). Each of the playbacks consisted of calls from at least six different recordings from the same individual (n = 8) recorded during three weeks prior to the start of the playbacks. Each group was subjected to playback calls from at least three different individuals.

Playback experiments were only conducted when no group member was on sentinel guard, to prevent any interference with other sentinel calls, and only when the group was foraging (i.e. more than 50% of the group members were foraging). If any of the conditions, including the absence of predators, were violated after the playback had been started, the playback was paused and resumed only after the majority of the group was back to normal foraging behaviour for a minimum of five minutes or the sentinel finished its guarding session. The speaker was kept at a height of 0.3–0.4 m as this has been shown to be a frequent height chosen by natural sentinels^44^ and also represented a good compromise to play back sentinel calls as well as close calls from the same height. The distance to the foraging test subject was no less than one meter but no more than two meters throughout the whole playback. In the case when a second playback was conducted in the same session, the second playback file differed in order of the five-minute playbacks and the calling individual. To analyse the behaviour of the test subject, behavioural focals were performed simultaneously to the playback of the different call types using a handheld Palm TX Tungsten (Palm Inc, 2005) with the focal program written in Cybertracker (Cybertracker Conservation 2013 version 3.376).

### Analysis of sound recordings and behavioural focals

**Figure 1:**
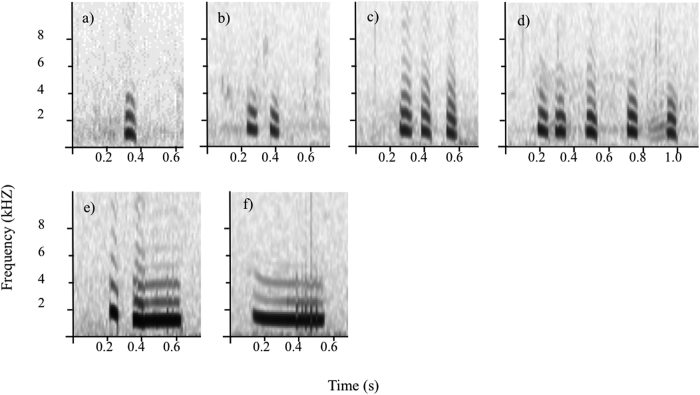
Spectrograms of the six sentinel call types divided into: i) short note calls: (**a**) single note call, (**b**) double note call, (**c**) triple note call; ii) long calls: (**d**) multiple note call, (**e**) di-drrr call, and (**f**) wheek call.

**Figure 2:**
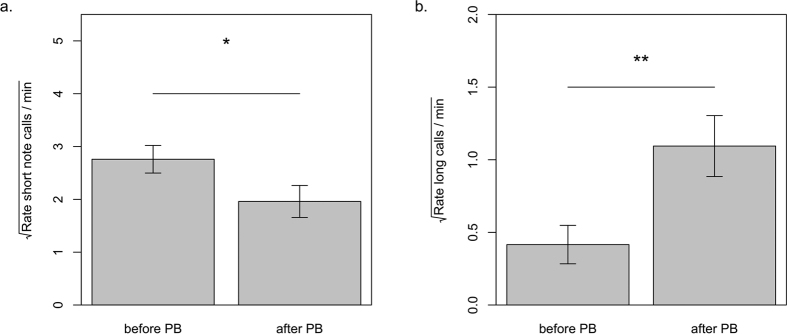
Influence of increased predation risk on production rates of the two categories of sentinel calls. Rates of (**a**) short note calls and (**b**) long calls two minutes before and 30 seconds after the playback (PB) of an alarm call. Shown are the transformed values of the mean and SE used for the LMM with asterixes indicating significance levels (*p < 0.05; **p < 0.01).

**Figure 3:**
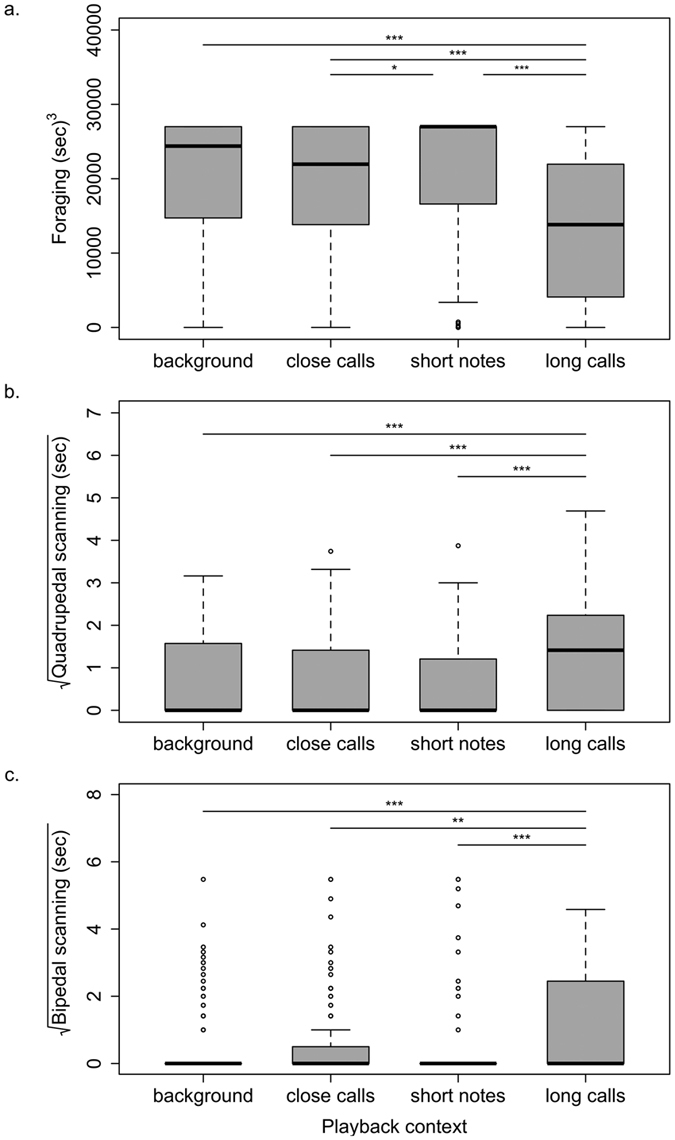
Influence of different sentinel call and control categories on foraging and vigilance behaviour. Time meerkats spent (**a**) foraging, (**b**) quadrupedal scanning and (**c**) on bipedal scanning during the different types of playbacks (calming calls, close calls and warning calls). Shown are the transformed values of the mean and SE used for the LMM with asterixes indicating significance levels (*p < 0.05; **p < 0.01; ***p < 0.001).

Sound recordings of naturally occurring sentinel sessions with a minimum duration of one minute were analysed using Cool Edit Pro (Syntrillium Software Corporation). In line with other studies on call combinations^30^,^45^, for a call to be classified as a call combination (i.e. “double note”, “triple note” or “multiple note”) the silence interval within the call combination had to be shorter than the silence interval to the previous and the following call. Work on meerkat call combination showed that the silence interval between two calls is almost 20 times longer than the silence interval within call combinations^30^, enabling call categorization of each call, by visual and audio inspection, as one of the six already described sentinel calls^23^ (Fig. 1). To quantify the immediate and short-term effect of alarm calls being played back, we analysed the call rates of the two different sentinel categories during two minutes prior to the playback of an alarm call and 30 seconds as well as five minutes afterwards separately.

For the analysis of the behavioural focals conducted during the sentinel playbacks, we focused on the behaviours recorded during 30 seconds after the occurrence of a call, rather than analysing the whole five minutes of the focal. Furthermore, because call rates of the different sentinel playback contexts (i.e. short note sentinel calls, long sentinel calls and close calls) differed according to their natural frequencies, we randomly selected four calls of every playback context to be analysed by using the sample function in R. As foraging behaviour we pooled all the different food-related behaviours, including foraging (digging in a hole for prey), scrabbling (scratching at multiple small holes or surface while steadily moving), processing (processing food items in sand, or chewing off tail of scorpions, etc.) and eating. Regarding the alert-related behaviours, we focused on two types of vigilance behaviour: quadrupedal scanning of surroundings, which is common and usually of short duration and bipedal scanning of surroundings, which is less frequent and of longer duration.

### Statistical analysis

All statistical analyses were done using R, release GUI 2.1 (R Development Core Team 2015). For all analyses linear mixed models^46^ were used in order to determine the relationship between call rate of sentinels or length of behaviours and the parameters of interest. To analyse changes in call rate of the sentinel after the playback of alarm calls, we used the rate of short note and long calls respectively as response variables and the relative timing of the playback (i.e. before and after the playback) as fixed effect. For the second part, when we analysed the behavioural responses of foraging group members to different calls, duration of each of the three focal behaviours (foraging, quadrupedal vigilance and bipedal vigilance) were used in three separate models and playback context (short note calls, long calls, close calls and background noise) was used as fixed effect. All the models contain individual identity nested in group identity as a random effect to control for differences in call rate between different individuals as well as between different groups. To determine whether alarm calls or the playback context had a significant effect on the response variable, likelihood ratio tests (LRT, v) were used to compare whether the model with the fixed effect included differs significantly from the same model with the fixed effect excluded^47^. To determine the fit of the linear mixed models we examined the model diagnostic plots and predictor variables were transformed where assumptions of the models were not met. Whenever the explanatory variable consisted of more than two categories multiple comparison test with manually set contrasts (multcomp function in R) were used to compare the different categories not specified by the intercept^48^.

### Ethical note

All research was conducted under the permission of the ethical committee of Pretoria University and the Northern Cape Conservation Service, South Africa (Permit number: EC011-10). All methods were carried out adhering to the approved guidelines in this permit.

## Additional Information

**How to cite this article:** Rauber, R. and Manser, M. B. Discrete call types referring to predation risk enhance the efficiency of the meerkat sentinel system. *Sci. Rep.* **7**, 44436; doi: 10.1038/srep44436 (2017).

**Publisher's note:** Springer Nature remains neutral with regard to jurisdictional claims in published maps and institutional affiliations.

## Figures and Tables

**Table 1:** LMM model outputs comparing the behavioural responses of foraging test subjects to the playbacks of the different call types (short note calls, close calls and long calls) to background noise (t-values).

Behaviour	Playback comparison		t/z	df	P
Foraging	Background	Close calls	0.86	3362	0.39
	Background	Short notes	1.14	3362	0.26
	Background	Long calls	−5.19	3376	<0.001***
	Short notes	Close calls	−2.01	—	0.045*
	Short notes	Long calls	−6.35	—	<0.001***
	Close calls	Long calls	−4.34	—	<0.001***
Quadrupedal scanning	Background	Close calls	0.53	395	0.59
	Background	Short notes	−0.06	395	0.29
	Background	Long calls	4.05	397.2	<0.001***
	Short notes	Close calls	1.55	—	0.123
	Short notes	Long calls	4.95	—	<0.001***
	Close calls	Long calls	3.43	—	<0.001***
Bipedal scanning	Background	Close calls	0.47	394.7	0.47
	Background	Short notes	−1.11	394.7	0.27
	Background	Long calls	3.1	395.9	<0.001***
	Short notes	Close calls	1.6	—	0.11
	Short notes	Long calls	4.86	—	<0.001***
	Close calls	Long calls	3.29	—	0.001**

Multiple comparison post-hoc tests showing the remaining comparisons among call types (z-values).
